# Long-Term Cognitive Impairments and Pathological Alterations in a Mouse Model of Repetitive Mild Traumatic Brain Injury

**DOI:** 10.3389/fneur.2014.00012

**Published:** 2014-02-04

**Authors:** Jian Luo, Andy Nguyen, Saul Villeda, Hui Zhang, Zhaoqing Ding, Derek Lindsey, Gregor Bieri, Joseph M. Castellano, Gary S. Beaupre, Tony Wyss-Coray

**Affiliations:** ^1^Department of Neurology and Neurological Sciences, Stanford University School of Medicine, Stanford, CA, USA; ^2^Center for Tissue Regeneration, Repair and Restoration, VA Palo Alto Health Care System, Palo Alto, CA, USA

**Keywords:** mild traumatic brain injury, long-term, neurobehavior, bioluminescence, astrogliosis

## Abstract

Mild traumatic brain injury (mTBI, also referred to as concussion) accounts for the majority of all traumatic brain injuries. The consequences of repetitive mTBI have become of particular concern for individuals engaged in certain sports or in military operations. Many mTBI patients suffer long-lasting neurobehavioral impairments. In order to expedite pre-clinical research and therapy development, there is a need for animal models that reflect the long-term cognitive and pathological features seen in patients. In the present study, we developed and characterized a mouse model of repetitive mTBI, induced onto the closed head over the left frontal hemisphere with an electromagnetic stereotaxic impact device. Using GFAP-luciferase bioluminescence reporter mice that provide a readout of astrocyte activation, we observed an increase in bioluminescence relative to the force delivered by the impactor after single impact and cumulative effects of repetitive mTBI. Using the injury parameters established in the reporter mice, we induced a repetitive mTBI in wild-type C57BL/6J mice and characterized the long-term outcome. Animals received repetitive mTBI showed a significant impairment in spatial learning and memory when tested at 2 and 6 months after injury. A robust astrogliosis and increased p-Tau immunoreactivity were observed upon post-mortem pathological examinations. These findings are consistent with the deficits and pathology associated with mTBI in humans and support the use of this model to evaluate potential therapeutic approaches.

## Introduction

Traumatic brain injury (TBI) is a major global health concern, and the CDC has estimated that over 1.5 million people experience a TBI each year in the US. Clinically, mild traumatic brain injury (mTBI, also referred to as concussion) accounts for approximately 80% of all TBIs, the majority of which result from closed head, concussive impact injuries (1). In military populations, mTBI caused by non-blast concussive impact and/or primary blast events has become the signature injury in the conflicts in Iraq and Afghanistan (2), with an estimated 12–35% of soldiers experiencing a mTBI during their deployment (3). While most mTBI patients recover without significant long-term consequences, 7–30% (even up to 60% in some reports) of individuals are estimated to suffer from a post-concussive syndrome that comprises physical, cognitive, and emotional symptoms (4, 5). These long-lasting symptoms include memory impairments, difficulty in concentration, depression, apathy, and anxiety (4, 5). Because these symptoms are usually observed in the absence of significant structural damage, patients sustaining mTBI are difficult to diagnose (6), and routine clinical and laboratory evaluations of mTBI patients often fail to show clear morphological brain defects.

Recently, the consequences of repetitive mTBI from multiple concussions have become of particular concern for individuals engaged in certain sports or in military operations because they are at high risk of repeated concussion (7, 8). Military personnel often have several mTBI exposures over the course of their lives and possibly within single deployments (9, 10). Epidemiological studies revealed that about 60% of retired professional football players sustained at least one concussion during their careers and approximately 25% experienced repeated injuries (11). Recurrent brain injuries, even when mild, may interfere with neuropsychological recovery (9, 12). Therefore, repetitive mTBI has been associated with greater severity of symptoms, with longer recovery time, and with earlier onset of age-related memory deficits and dementia (13). Repeated concussions have also been associated with chronic traumatic encephalopathy (CTE), a neurodegenerative disorder with progressive impairments of memory and cognition, as well as depression, anxiety, and motor abnormalities (14–16).

Our understanding of the mechanisms of these behavioral deficits is still largely incomplete. Animal models would certainly facilitate a better understanding of the pathological and behavioral outcomes as a result of concussion (17–19). Therefore, there has been a recent focus in developing animal models of mTBI. A large number of animal models of mTBI have been developed and they have been effective in characterizing the pathological and behavioral changes after acute (i.e., single impact) mTBI. Current animal models of concussion have included mild to moderate versions of fluid-percussion impact (FPI), controlled cortical impact (CCI), and weight drop injury (20, 21). Although these models have demonstrated mild to moderate injury severity levels, most are not capable of mimicking true closed-head concussive injury. Furthermore, most of the current mTBI models have been reported to produce some degree of immediate or short-term behavioral deficits; however, it is not clear whether these deficits are long-lasting since few of the published studies made long-term observations beyond 1 month (17, 18). Therefore, our goal of this study was to develop a clinically relevant closed-head injury of repetitive mTBI that results in long-term behavioral and pathological alterations modeling mild brain injury in humans. To establish injury parameters, we first employed bioluminescence imaging in reporter mice expressing luciferase under the control of a GFAP promoter (GFAP-luc mice) (22, 23). Bioluminescence imaging of GFAP-luc mice enables us to test a relatively large number of injury conditions in a medium-throughput manner by following astrogliosis and neural injury in the same mice throughout the course of injury. We then implemented the injury parameters established in the reporter mice and induced a repetitive mTBI in wild-type C57BL/6J mice. To further validate the long-term cognitive and pathological effects of the injury model, we performed behavioral testing and post-mortem pathological examinations of brain tissue.

## Materials and Methods

### Animals

The experiments were approved by the Institutional Animal Care and Use Committee of VA Palo Alto Health Care System, and were in accordance with the National Institutes of Health Guide for the Care and Use of Laboratory Animals. Two mouse lines were used: bioluminescence reporter mice expressing luciferase under the control of a GFAP promoter (GFAP-luc mice, FVB/N background) (Caliper Life Science, now part of Caliper Life Science, Hopkinton, MA, USA) (22, 23) and wild-type C57BL/6J mice (The Jackson’s Laboratory, Bar Harbor, ME, USA). Bedding, nesting material, food, and water were provided *ad libitum*. Ambient temperature was controlled at 20–22°C with 12-h light/12-h dark cycles. Mice were 2–3 months of age at the beginning of experiments. All behavioral testing was performed in isolated behavior rooms.

### A mouse model of repetitive closed-head mTBI

**Figure 1:**
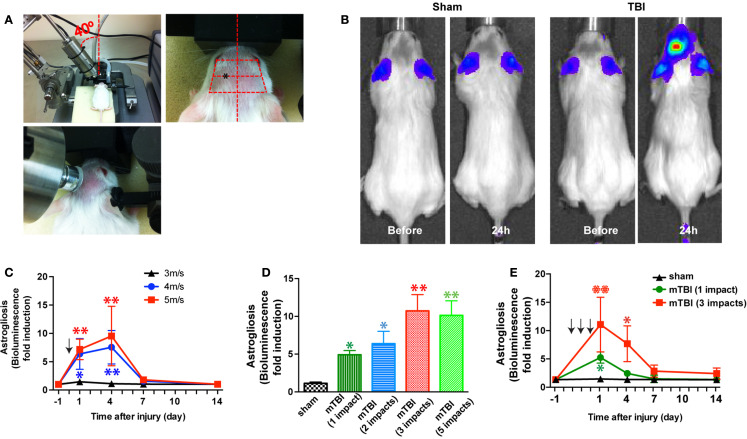
**A mouse model of closed-head mTBI using an electromagnetically controlled stereotaxic impact device and injury severity-dependent activation of a reporter gene**. **(A)** A mouse in the stereotaxic frame with the stereotaxic electromagnetic impactor aligned above the head [**(A)**, left panel] and with the impactor tip touching the surface of the head [**(A)**, middle panel]. The tip was fully extended and lowered at a 40° angle until it touched the head at the defined location (over the left frontotemporal lobe) [**(A)**, right panel]. **(B)** An mTBI induces reporter gene activation in GFAP-luc mice. Representative images show increased bioluminescence signals over baseline in the head after injury (right panel), but not in sham treated mice (left panel). **(C)** GFAP-luc mice (male, 2–3 months old, body weight 25.63 ± 3.74 g) were randomly assigned to receive a single impact (shown by the arrow) at different speeds (3, 4, or 5 m/s respectively) but identical distance and dwell time as above. Bioluminescence is expressed as fold induction over baseline. *n* = 5 mice/group. Mean ± SD; *P < 0.05; **P < 0.01; two-way ANOVA and *Bonferroni* test. **(D)** GFAP-luc mice (female, 2–3 months old, body weight 20.45 ± 2.13 g) were randomly assigned to receive different number of impacts. The impact was induced at a speed of 4.0 m/s with a dwell time of 0.2 s. Bioluminescence as a function of astrocyte activation was measured 24 h after last impact and expressed as fold induction over baseline (measured 1 day before injury for each mouse). *n* = 5 mice/group. Mean ± SD; *P < 0.05; **P < 0.01; ANOVA and *Bonferroni* test. **(E)** GFAP-luc mice (female, 2–3 months old, body weight 20.31 ± 2.37 g) were randomly assigned to receive three impacts (repetitive, impact–impact–impact, once a day for 3 days, shown by the arrows), one impact (single, sham–sham–impact), and one sham (sham, sham–sham–sham) procedures. The impact was induced by moving down the stereotaxic device 3.0 mm, at a speed of 4.0 m/s with a dwell time of 0.2 s. Bioluminescence as a function of astrocyte activation was measured and expressed as fold induction over baseline (measured 1 day before injury for each mouse). *n* = 5 mice/group. Mean ± SD; *P < 0.05; **P < 0.01; two-way repeated measures ANOVA and *Bonferroni* test.

Closed-head injury was induced using a Benchmark Stereotaxic Impactor (MyNeurolab, St. Louis, MO, USA) with modifications. The Benchmark Stereotaxic Impactor is an electromagnetic stereotaxic impact device capable of producing consistent, graded CCI injuries in adult mice with stereotaxic control of impact location and depth at high velocities (24). Recently it was modified to produce repetitive closed-skull TBI in mice (25). We mounted an actuator on a stereotaxic frame (David Kopf Instruments, Tujunga, CA, USA) at a 40° angle (Figure 1A, left panel). A rubber tip (25) or self-adhesive bumper (3M, St. Paul, MN, USA) was mounted onto a customized impact probe tip (9 mm in diameter). Mice were anesthetized with isoflurane (2.5% induction, 1.0% maintenance) and the scalp was shaved. Mice were placed in the stereotaxic frame and secured in prone position in a customized foam mold, with isoflurane delivered by a gas anesthesia mask (Stoelting, Wood Dale, IL, USA) (Figure 1A, left panel). An ear bar (with the non-pointed end touching the skin) (Stoelting, Wood Dale, IL, USA) was used to assist to position the head. The probe tip was fully extended and lowered until the vertex of the bumper touched the scalp (Figure 1A, middle panel). The stereotaxic manipulator was adjusted so that the vertex of the bumper impacted the scalp at a consistent point in relation to the eye and ear of injury side of the head (Figure 1A, right panel). The center of impact corresponds to the following coordinates: from Bregma: AP 1.2 mm (1.2 mm anterior to the bregma), ML 4.2 mm (4.2 mm lateral to the midline), according to the mouse brain atlas (26). To induce an impact, the tip was retracted automatically. The ear bar was gently removed to allow the head to move with little or no restraint upon impact. The stereotaxic device was then moved down, and the electromagnetic device was triggered, driving the tip into the head at a speed and a dwell time set from the electronics control box. The size and shape of the probe tip and bumper, the distance that stereotaxic device is moved down (impact depth), the speed the probe tip travels (impact speed), and the dwell time can be varied to achieve concussions of different severities. In addition, the location and the angle at which the probe tip impacted the head (impact location and angle) can be adjusted through the stereotaxic manipulator. After impact, the mice were allowed to recover from anesthesia on a warming pad and then returned to their home cages. For repetitive injuries, identical impact procedures were performed at an interval of 24 ± 1 h. For sham injuries, the same procedure was performed except that the impact device was discharged in the air; the handling of the mice and duration of anesthesia were identical for both mTBI and sham procedures (24, 25).

In this study, we chose to vary impact speeds (3–5 m/s) for the injury phase while keeping other parameters (impact depth: 3 mm; dwell time: 0.2 s; impact probe tip: 9 mm; and impact angle: 40°) consistent. Similar parameters have been used to induce mTBI in CCI or closed-skull concussions (24, 25). A total of 106 mice (included both lines) were used in this study. There were no immediate fatalities with these settings. Subcutaneous hemorrhages were observed in GFAP-luc mice impacted with 5 m/s of impact speed (10%) and with repetitive impacts (25%). These mice with excluded from behavioral and pathological analyses. Subcutaneous hemorrhages or skull fractures were not observed in C57BL/6J mice after three impacts of repetitive injury at 4 m/s.

### *In vivo* bioluminescence imaging

Bioluminescence emitted from the brain of GFAP-luc mice was detected with the *In vivo* Imaging System (IVIS Spectrum; Caliper Life Science, Alameda, CA, USA) as previously described (22, 27, 28). Briefly, mice were injected intraperitoneally with 150 mg/kg d-luciferin 10 min before imaging and anesthetized with isoflurane during imaging. Photons emitted from living mice were acquired as photons per second per square centimeter per steradian using LIVINGIMAGE software (version 4.0) and integrated over 3 min. For bioluminescence quantification, a region of interest was manually selected over the head and kept constant for all experiments; the signal intensity was converted into photons per seconds per square millimeter per steradian. For longitudinal comparison of bioluminescence, baseline imaging was performed 24 h before impact injury and bioluminescence was expressed as fold induction over baseline levels for each mouse.

### Smart-Homecage monitoring

The Smart-Homecage, developed by AfaSci, Inc.^1^ (Redwood City, CA, USA), is a home cage behavior monitoring system (29). This system is composed of infrared (IR) matrices for activity, position, and locomotion detection. A fresh cage was inserted into the Smart-Homecage platform. The mouse was placed in the cage and allowed to explore the cage for 5 min. Parameters of exploratory behavior, such as travel distance and rearing were calculated automatically by CageScore software.

### Rota-Rod testing

Mice were trained and tested on an accelerating Rota-Rod (Ugo Basile North America Inc., Collegeville, PA, USA) for motor strength and coordination. The test procedure was performed according to the SOP developed by the EUMORPHIA consortium^2^. There is no training period prior to the test phase as this actually improves the reproducibility and strain ranking effects across centers (30). The Rota-Rod was set to accelerate from 5 to 30 rpm during a test period of 5 min. Mice were tested three times with an inter-trial interval of 20 min, and the latency to fall onto the transducer platform was automatically recorded. The average latency of the three trials was calculated for the analysis.

### DigiGait analysis

The DigiGait System (Mouse Specifics, Boston, MA, USA) is a non-invasive method for quantitative comparison of gait dynamics. Each mouse was placed on a transparent belt of a treadmill enclosed by a plastic scaffold. The speed of the belt was set at 20 cm/s for all mice. The mouse was imaged ventrally by a high-speed camera, which captured the dynamics of the paws and corresponding limbs while walking on the belt. The stride length and other spatial and temporal gait indices for each limb were analyzed automatically with the DigiGait Imaging System.

### Elevated zero maze testing

The elevated zero maze was used to test for unconditioned anxiety-like behaviors (31). The maze is a circular platform (outer diameter 46 cm, width 5.5 cm) that was elevated 40 cm above the floor. It consists of two walled (white Plexiglas) sectors separated by two open sectors of equal length. Each test session was started by placing the mouse in one of the two open sectors facing a closed sector. The mouse was allowed to freely explore for 5 min. Activity was recorded using a video-camera placed above the maze and TopScan software (Clever Sys., Inc., Reston, VA, USA). Total path traveled in the different sectors, percent of time spent in the open and closed sectors, and number of open sector entries were automatically analyzed by TopScan software. The mouse was then returned to its home cage and the maze was cleaned with a 70% ethanol between animals.

### Radial arm water maze

Spatial learning and memory was assessed using the radial arm water maze (RAWM) paradigm following the exact protocol described previously (32). The goal arm location containing a platform remained constant throughout the training and testing phase, while the start arm was changed during each trial. On day 1 during the training phase, mice were trained for 15 trials, with trials alternating between a visible and hidden platform. On days 2 and 3 during the testing phase, mice were tested for 15 trials with a hidden platform. Entry into an incorrect arm was scored as an error, and errors were averaged over training blocks (three consecutive trials).

### Y-maze

The Y-maze is made of solid white plastic and consisted of two symmetrical arms and one longer arm at 120° angles (longer arm, 20.7 cm length × 12.7 cm height × 7.62 cm width; equal arms, 15.24 cm length × 12.7 cm height × 7.62 cm width) (33). At the beginning of trials, mice were placed in the end of the longer arm and allowed to freely explore the three arms for 5 min. Arm entry was defined as having all four limbs inside an arm. The maze was cleaned with 70% ethanol between animals and before the first animal to eliminate traces of odor. The number of arm entries and the number of triads were recorded in order to calculate the alternation percentage, which was calculated by dividing the number of triads by the number of possible alternations multiplied by 100. A triad was defined as a set of consecutive arm entries (34).

### Contextual fear conditioning

Contextual fear conditioning was performed following the previously published procedures (32) with minor modifications. In this task, mice learned to associate the environmental context (fear conditioning chamber) with an aversive stimulus (mild foot shock; unconditioned stimulus, US) enabling testing for hippocampal-dependent contextual fear conditioning. As contextual fear conditioning is hippocampus and amygdala-dependent, the mild foot shock was paired with a light and tone cue (conditioned stimulus, CS) in order to also assess amygdala-dependent cued fear conditioning. Conditioned fear was displayed as freezing behavior. Specific training parameters are as follows: tone duration is 30 s; sound level is 70 dB, 2 kHz; shock duration is 2 s; and intensity is 0.6 mA. More specifically, on the first day of testing (training), each mouse was placed in a fear conditioning chamber and allowed to explore for 2 min before delivery of a 30-s tone (70 dB) ending with a 2-s foot shock (0.6 mA). Two minutes later, a second CS–US pair was delivered. Shocks were delivered through the grid floor and were controlled by FreezeScan software (Clever Sys., Inc., Reston, VA, USA). On the second day, each mouse was placed in the same fear conditioning chamber containing the same context as the training day, but without administration of a CS or foot shock. Freezing was analyzed for 1–3 min. To analyze cued freezing behavior, the mice were placed in a new context 1 h later that contained a different odor (3% acetic acid), cleaning solution, floor texture, chamber walls, and shape. Animals were allowed to explore for 2 min before being re-exposed to the CS. Freezing was analyzed for 1–3 min. Freezing was measured using a FreezeScan video tracking system and software (Clever Sys., Inc., Reston, VA, USA).

### Tissue processing

Mice were anesthetized with 400 mg/kg chloral hydrate (Sigma-Aldrich) and transcardially perfused with 0.9% saline (22, 28). Brains were removed and postfixed in phosphate-buffered 4% paraformaldehyde, pH 7.4, at 4°C for 48 h and sectioned at 40 μm with a sliding microtome 2010 (Leica, Allendale, NJ, USA). The sections were collected serially in 12 tubes and stored in cryoprotective medium (22, 28).

### Cresyl violet staining

Brain sections (every 12th section) were mounted on Superfrost plus slides (Fisher Scientific, Pittsburgh, PA, USA), air-dried, rehydrated, stained with 0.02% Cresyl Violet (Sigma) in acetate buffer (pH 3.2), then dehydrated through a series of alcohols, cleared in xylene, and coverslipped (28). Neuronal damage/loss was assessed based on the appearance of gaps or thinning and disappearance of the Nissl substance in the CA1 and CA3 pyramidal cell layers. The lesion area was quantified with MetaMorph Imaging software (Molecular Devices, Downingtown, PA, USA).

### Immunohistochemistry, light microscopy, and image analysis

Immunohistochemistry was performed on free-floating sections (every 12th section) following standard procedures (22, 28). Primary antibodies were against: cleaved caspase-3 (1:1000, Cell Signaling Technology, Danvers, MA, USA), GFAP (1:1000, Dako, Carpinteria, CA, USA) (35), p-Tau (Ser202/Thr205, AT8) (1:1000, Pierce, Rockford, IL, USA) (36), and p-CREB (Ser 133) (1:1000, Millipore, Billerica, MA, USA) (37). After overnight incubation, primary antibody staining was revealed using biotinylated secondary antibodies and the ABC kit (Vector, Burlingame, CA, USA) with Diaminobenzidine (DAB, Sigma-Aldrich). Photographs were acquired using a BX51 microscope (Olympus) and a SPOT Flex shifting pixel CCD camera with SPOT Advanced software (SPOT Imaging Solutions, Sterling Heights, MI, USA) under identical conditions and settings. The immunoreactivity was quantified as the percent area covered by ImageJ software (NIH, Bethesda, MD, USA). Our initial observations revealed pathological changes mainly from 0.98 to −2.06 mm to Bregma, and we therefore restricted our quantification to sections covering that region. This resulted typically in six and seven sections separated roughly by 12 μm × 40 μm, with half of the sections containing hippocampus. We therefore performed image analysis in five sections/animal for cortex and corpus callosum, and three sections/mouse for hippocampus. The images were first converted to eight-bit gray-scale images and then converted into binary positive/negative images by thresholding held constant for all images in a given brain region. Percent area fraction covered by the threshold was determined by ImageJ. The average of values obtained from the three or five sections was used for each animal for statistical analysis.

### Data analysis

Data are presented as mean ± SD or mean ± SEM depending on the type of experiment. Statistical analysis of behavioral measurements was performed with GraphPad Prism software (version 6) using ANOVA (regular or repeated measures) and *Bonferroni post hoc* test or two-tailed Student’s *t*-test where appropriate. *P* < 0.05 was considered statistically significant.

## Results

In this study, we carried out two series of experiments: the first to investigate injury (impact intensity)-dependent responses in the GFAP-luc reporter mice (FVB/N genetic background) to establish injury parameters, and the second to investigate the behavioral and pathological outcome of repetitive closed-head concussive brain injury based on the above parameters in wild-type C57BL/6J mice.

### A mouse model of repetitive closed-head mTBI

In the first series of experiments we employed bioluminescence imaging, which enables us to follow astrogliosis and neural injury in the reporter mice expressing luciferase under the control of a GFAP promoter (GFAP-luc mice) (on FVB/N genetic background) (22, 23). Neuronal injury is closely tied to astrogliosis, and we and others have previously shown that bioluminescence intensity in the GFAP-luc mice correlates significantly with astrogliosis assessed by immunohistochemistry and with neural injury in brain injury models of excitotoxicity and experimental autoimmune encephalomyelitis (22, 23). In this study, we utilized this technique to take advantage of testing a relatively large number of injury conditions in a medium-throughput manner while studying the same mouse throughout the course of injury. In an initial experiment, a mild traumatic impact (impact speed: 4 m/s) to the head led to a reproducible, significant increase in bioluminescence in the brain (Figure 1B). Notably, no significant increase in bioluminescence was observed after a sham procedure, suggesting that the sham procedure caused negligible disturbance to the brain. To determine whether different degrees of injury cause different induction of bioluminescence signal, we impacted mice at different impact speeds (3, 4, and 5 m/s, respectively) and observed a dose-dependent increase of the bioluminescence signal (Figure 1C). To determine whether repetitive injury is cumulative, we compared the bioluminescence signals in mice receiving different numbers of impacts (impact speed: 4 m/s; interval: approximately 24 h) (Figure 1D). Twenty-four hours after the last impact, there was a significant increase in bioluminescence signal in all injury groups compared with the sham group. There appeared to be a dose-dependent increase in bioluminescence signals in mice receiving one to three impacts, but mice receiving five impacts did not show further increase in bioluminescence signals compared with those receiving three impacts (Figure 1D). We then compared the time course of bioluminescence signals in mice receiving a single impact with those receiving three impacts (Figure 1E). In the single-impact group, bioluminescence signals increased significantly at 24 h after impact (*P* < 0.05, compared with baseline), but deceased significantly at day 3 (*P* > 0.05, compared with baseline), and returned to pre-injury levels at day 7. The repetitive group showed significantly higher bioluminescence signals at 24 h after the third impact than the single-impact group (*P* < 0.01, compared with baseline and with the single-impact group). While the bioluminescence signals in the repeat-impact group decreased to the same degree as in the single group at day 3, they were significantly higher overall (*P* < 0.01, compared with baseline and with the single-impact group). At days 7 and 14, the bioluminescence signals in the repeat-impact group were higher than baseline and the single-impact group, but did not reach statistical significance (*P* > 0.05).

**Figure 2:**
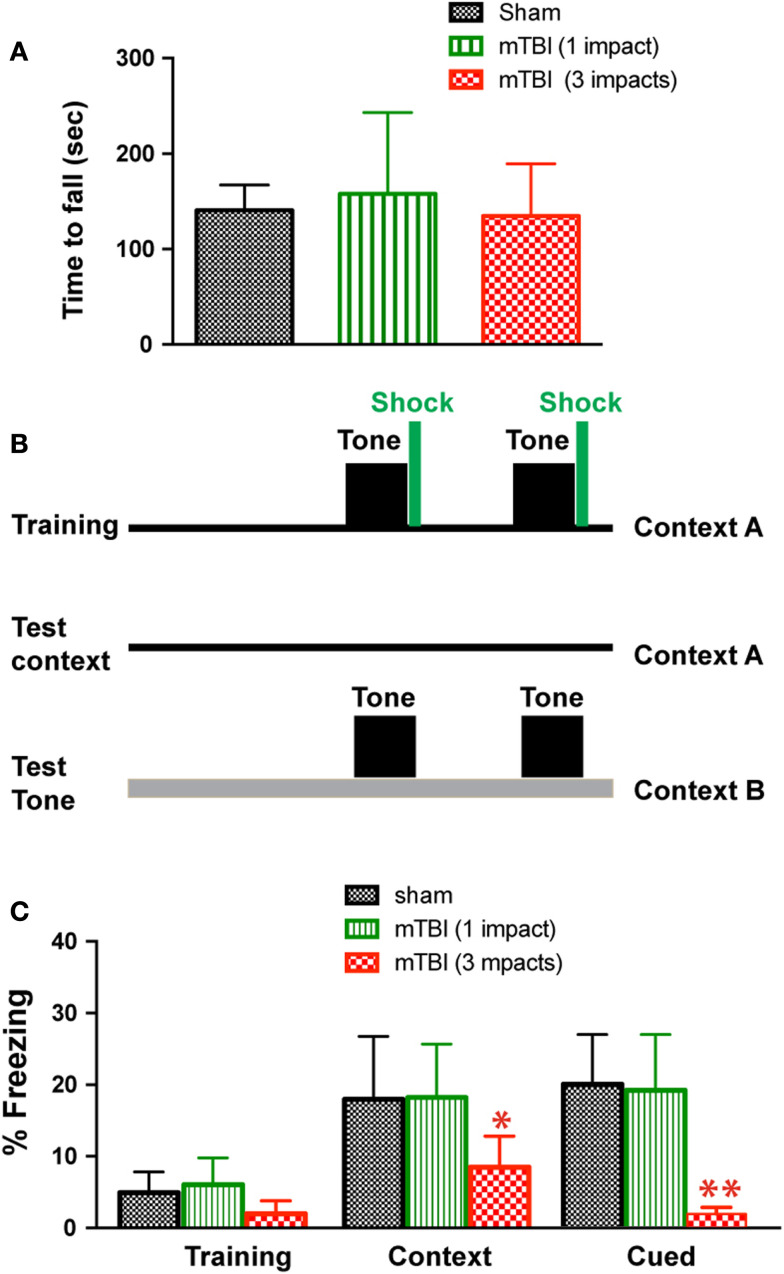
**Repetitive mTBI causes significant cognitive impairments 3 months after injury**. Three months after injury, the GFAP-luc mice shown in Figure 1E were tested for motor function using a Rota-Rod apparatus **(A)** and for learning and memory using fear conditioning **(B,C)**. **(A)** In the Rota-Rod test, latency to fall onto the transducer platform was automatically recorded. The average latency over the three trials was calculated for each mouse. No difference between the mTBI and sham groups were observed (mean ± SD, *P* > 0.05 by *t* test). **(B)** The test paradigm for fear conditioning. **(C)** The freezing behavior was recorded and analyzed. Mice exposed to mTBI (three impacts) showed reduced baseline freezing behavior, and context- and cue-related freezing behavior. *n* = 5 mice/group. Mean ± SD; *P < 0.05, **P < 0.01; ANOVA and *Bonferroni* test.

To determine whether mTBI in our model causes motor deficits, we performed Rota-Rod test. There was no significant difference between injured (one or three impacts) and sham groups (Figure 2A). To determine whether mTBI causes long-term cognitive deficits, we performed a fear conditioning test 3 months after injury (Figure 2B). During fear conditioning training, mTBI mice receiving three impacts exhibited reduced baseline freezing time (Figure 2C). Importantly, mTBI mice receiving three impacts demonstrated decreased freezing time during both cued and contextual memory testing compared with sham animals (*P* < 0.05). In contrast, mTBI mice receiving one impact did not differ significantly from sham mice in freezing behavior at baseline, nor did they differ significantly in cued or contextual memory (Figure 2C). Thus, repetitive mTBI in our close-head model produces consistent mild injuries, leading to long-term memory, and cognitive deficits. In addition, correlation analysis shows that bioluminescence signal in the GFAP-luc mice negatively correlates with freezing behavior in the fear conditioning test (*R* = −0.704, *P* = 0.012) and with post-mortem p-CREB immunoreactivity (*R* = −0.744, *P* = 0.007), suggesting that bioluminescence signal can be used as a surrogate marker in our mTBI paradigm.

### Repetitive closed-head mTBI caused cognitive deficits 6 months after injury in wild-type C57BL/6J mice

**Figure 3:**
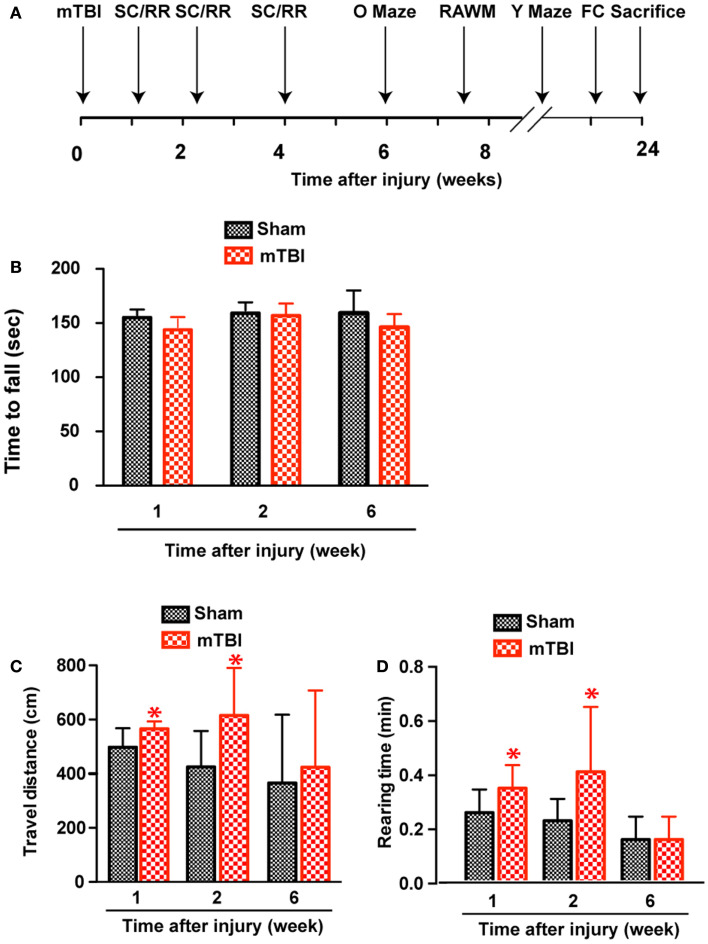
**Increased exploratory behavior after repetitive mTBI without significant motor defects**. Wild-type C57BL/6J mice (male, 3 months of age, body weight 24.14 ± 3.18 g) received three mild impacts (mTBI) or underwent sham procedures (sham). **(A)** Schematic showing schedule of behavioral tests employed in this study at the following time points after injury: SmartCage (SC) and Rota-Rod (RR), 1, 2, and 6 weeks; Elevated zero maze (O maze), 6 weeks; radial arm water maze (RAWM), 7–8 weeks; and Y-maze and fear conditioning (FC) 23–24 weeks. **(B)** Motor function was tested by a Rota-Rod apparatus. No difference between the mTBI and sham groups was observed at any time points. **(C,D)** Exploratory behavior was monitored for 5 min in the SmartCage. Mice exposed to mTBI traveled significantly longer distance **(C)** and reared significantly more **(D)** than the sham group at 1 and 2 weeks after injury. *n* = 8 (sham) and 15 (mTBI) mice/group. Mean ± SD; *P < 0.05, repeated measures ANOVA.

Based on the results of astrogliosis and the behavioral deficits observed in the reporter mice, the following injury parameters were selected: impact speed: 4 m/s; number of impacts: 3; and injury interval: 24 h. We induced repetitive mild brain injury and characterized the outcome in wild-type C57BL/6J mice, a mouse line more suitable for behavioral characterization and more commonly used in the field. We employed a battery of behavioral tests (Figure 3A) and performed post-mortem histopathological analysis to investigate whether the repetitive mTBI in our model is capable of producing impairments similar to what is observed in human mTBI patients.

### Increased exploratory behavior based on homecage monitoring

At weeks 1 and 2, mTBI mice showed significantly increased exploratory behavior during the first 5 min in a new environment, shown by increased travel distance and rearing behavior (Figures 3C,D). However, no significant difference was found at 6 weeks after injury, suggesting that this effect recovered. In addition, there was no difference in either travel distance or rearing behavior over 60-min monitoring (not shown), suggesting mTBI did not cause significant motor deficits.

### No significant motor deficits after repetitive mTBI

Absence of significant motor deficits in these mice was further confirmed using Rota-Rod (Figure 3B), and the DigiGait analysis system (Mouse Specifics, Inc.), which offers an automated analysis of a large number of gait parameters. There was no consistent difference between sham and mTBI mice in any of the parameters measured, including stride length, stride width, stride frequency, and stride length variability (Table 1).

**Table 1:** **Gait parameters 2 weeks after injury**.

	Left front		Left hind		Right front		Right hind	
	Sham	mTBI	Sham	mTBI	Sham	mTBI	Sham	mTBI
Swing (s)	0.111 ± 0.005	0.122 ± 0.004	0.110 ± 0.002	0.107 ± 0.004	0.115 ± 0.007	0.117 ± 0.003	0.097 ± 0.004	0.106 ± 0.003
% Swing/stride	38.16 ± 1.07	39.51 ± 0.98	35.59 ± 0.93	35.23 ± 1.44	39.74 ± 0.09	38.81 ± 0.72	35.29 ± 1.05	35.67 ± 0.86
Braking (s)	0.087 ± 0.002	0.096 ± 0.005	0.038 ± 0.004	0.041 ± 0.003	0.088 ± 0.004	0.086 ± 0.005	0.043 ± 0.002	0.046 ± 0.002
% Braking/stride	30.47 ± 0.57	31.20 ± 1.68	13.65 ± 1.37	13.33 ± 1.02	30.76 ± 1.60	28.83 ± 1.96	15.96 ± 1.14	15.61 ± 0.83
Propel (s)	0.092 ± 0.009	0.091 ± 0.005	0.144 ± 0.009	0.158 ± 0.007	0.085 ± 0.005	0.098 ± 0.006	0.135 ± 0.009	0.145 ± 0.004
% Propulsion/stride	31.37 ± 1.99	29.26 ± 1.83	50.76 ± 2.16	52.426 ± 1.28	29.53 ± 0.87	32.37 ± 1.86	48.76 ± 1.53	48.72 ± 0.87
Stance (s)	0.180 ± 0.009	0.186 ± 0.003	0.181 ± 0.007	0.198 ± 0.007	0.173 ± 0.006	0.185 ± 0.003	0.178 ± 0.007	0.192 ± 0.005
% Stance/stride	61.4 ± 1.073	60.49 ± 0.083	64.41 ± 0.093	64.77 ± 1.44	60.26 ± 0.090	61.19 ± 0.72	64.71 ± 1.05	64.33 ± 0.86
Stride length (cm)	5.80 ± 0.23	6.17 ± 0.11	5.63 ± 0.16	6.11 ± 0.15	5.74 ± 0.25	6.03 ± 0.08	5.53 ± 0.21	5.95 ± 0.15
Stride (s)	0.290 ± 0.011	0.309 ± 0.005	0.281 ± 0.008	0.305 ± 0.007	0.288 ± 0.012	0.302 ± 0.004	0.276 ± 0.010	0.298 ± 0.007
Stance width (cm)	1.70 ± 0.06	1.67 ± 0.04	2.56 ± 0.07	2.49 ± 0.04	NA	NA	NA	NA
Stance/swing (ratio)	1.66 ± 0.08	1.56 ± 0.07	1.81 ± 0.07	1.89 ± 0.12	1.53 ± 0.05	1.59 ± 0.05	1.84 ± 0.08	1.82 ± 0.07

### No anxiety-like behavior in the elevated zero maze tests

**Figure 4:**
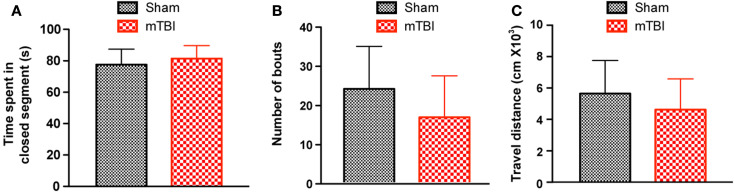
**No significant difference was observed in the zero maze test 6 weeks after repetitive brain injury**. Wild-type C57BL/6J mice (male, 3 months of age) received three mild impacts (mTBI) or underwent sham procedures (sham), and were assessed for anxiety using the elevated zero maze. The results were expressed as time spent in the closed segment **(A)**, number of bouts **(B)**, and travel distance **(C)**. *n* = 8 (sham) and 15 (mTBI) mice/group. Mean ± SD.

To investigate whether mTBI mice show anxiety-like behavior we employed an elevated zero maze test (31) and observed no differences between mTBI and sham groups. Specifically, the mTBI mice spent similar amounts of time in the closed arms of the maze (Figure 4A). In addition, the number of bouts and the distance moved in the entire maze were not significantly different between the two groups (Figures 4B,C). Thus, mTBI mice did not display unconditioned anxiety-like behaviors.

### Impaired spatial learning and memory 2 months after repetitive mTBI

**Figure 5:**
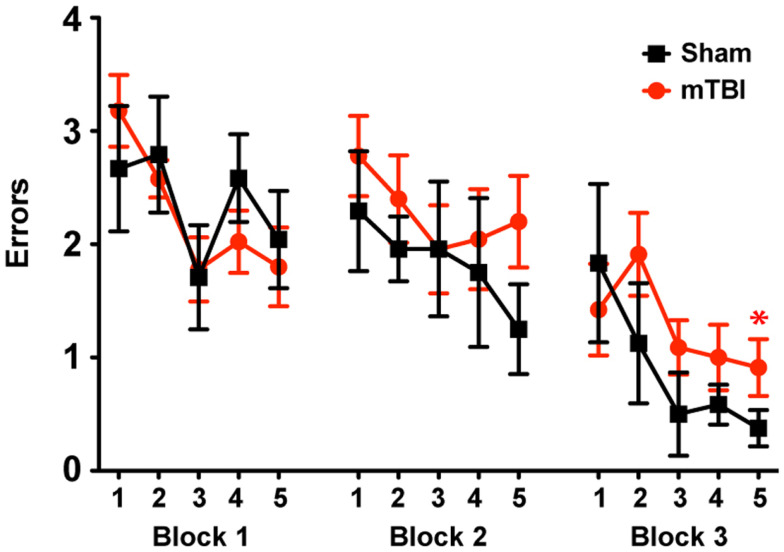
**Repetitive mTBI causes significant cognitive impairments 2 months after injury**. Wild-type C57BL/6J mice (male, 3 months of age) received three mild impacts (mTBI) or underwent sham procedures (sham). Spatial learning and memory was assessed using the radial arm water maze (RAWM) paradigm 2 months after injury. On day 1 during the training phase, mice are trained for 15 trials, with trials alternating between visible and hidden platforms. On day 2 during the testing phase, mice are tested for 15 trials with a hidden platform. Entry into an incorrect arm is scored as an error, and the errors are averaged over training blocks (three consecutive trials). Learning and memory deficits were quantified as the number of entry arm errors made prior to finding the target platform. *n* = 8 (sham) and 15 (mTBI) mice/group. Mean ± SEM; *P < 0.05, repeated measures ANOVA.

To determine whether the repetitive mTBI in our model led to cognitive impairments in mice we assessed hippocampal-dependent learning and memory using the RAWM paradigm (2 months after injury). All mice showed similar spatial learning capacity during the training phase regardless of treatment. However, by the end of the testing phase animals exposed to repetitive mTBI exhibited impaired learning and memory deficits, committing significantly more errors in locating the target platform than sham animals (Figure 5).

### Cued and contextual memory deficits 6 months after repetitive mTBI

**Figure 6:**
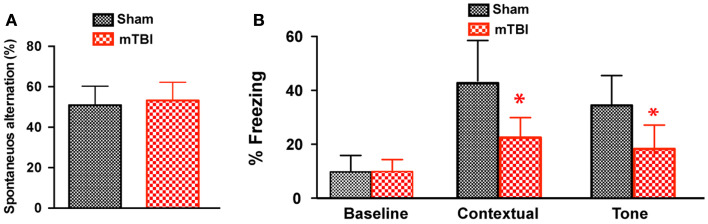
**Repetitive mTBI causes significant cognitive impairments 6 months after injury**. Wild-type C57BL/6J mice (male, 3 months of age) received three mild impacts (mTBI) or underwent sham procedures (sham). Cognitive function was assessed using the Y-maze **(A)** and contextual fear conditioning **(B)** 6 months after injury. **(A)** In the Y-maze test for working memory, there was no difference in spontaneous alternations between mTBI and sham mice. **(B)** In the fear conditioning test, no significant statistical difference was apparent in baseline freezing, however, mTBI mice showed significantly impaired context and cued memory compared with sham animals. *n* = 8 (sham) and 15 (mTBI) mice/group. Mean ± SD. *P < 0.05; by *t* test.

To determine whether the repetitive mTBI in our model led to cognitive impairments at a later time point, we performed Y-maze and contextual fear conditioning tests at 6 months after injury. There was no difference in spontaneous alternation in the Y-maze test (Figure 6A). In the fear conditioning test, both the sham and mTBI animals exhibited similar baseline freezing behavior during training (Figure 6B). However, the mTBI group performed significantly worse in the contextual test than the sham group (Figure 6B), as shown by the significantly decreased freezing behavior. In addition, the mTBI group showed decreased freezing behavior in cued memory retrieval detected 24 h after training when re-exposed to the CS (tone and light) in a novel context (Figure 6B). Results from contextual and cued periods were consistent with our observations in FVB/N mice (Figure 2). Together, these data demonstrate that repetitive mTBI impairs learning and memory as long as 6 months after injury in our model.

### Prominent pathological alterations after repetitive mTBI

To determine if the observed behavioral deficits are associated with pathological changes (6 months after injury), we performed post-mortem pathological examinations. We first performed cresyl violet staining and did not observe contusions or obvious cell loss (for example, percent area covered was 76.97 ± 4.53 in CA3 in mTBI and 79.29 ± 5.46 in sham, mean ± SEM, *P* = 0.757 by *t* test). We then performed immunohistochemistry using antibodies against cleaved caspase-3 (marker of apoptotic cell death, but we observed only a few caspase-3 positive cells/section after mTBI, which was not significantly different from that of sham), GFAP (a marker of astrogliosis), p-Tau (a marker of impaired axonal transport and axonal damage), and p-CREB [a marker of activation of cAMP responsive element binding protein (CREB) pathway related to memory formation and retention].

### Strong astrogliosis after repetitive mTBI

**Figure 7:**
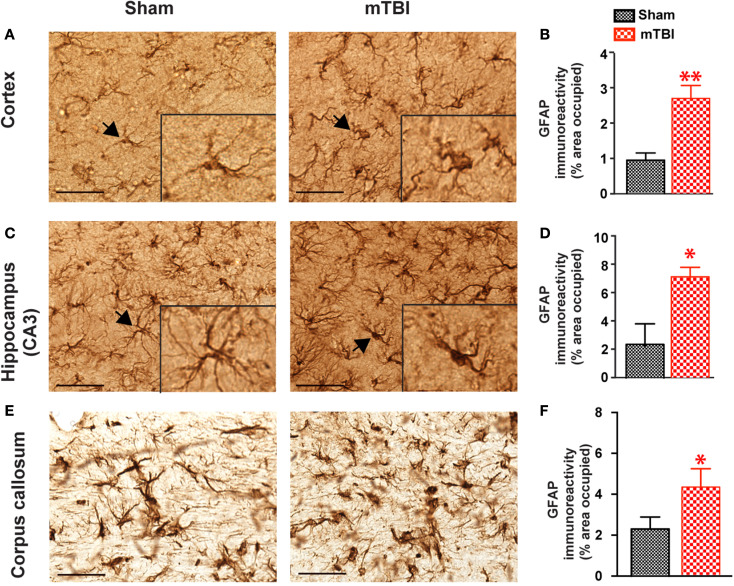
**Significant astrogliosis after repetitive mTBI**. Wild-type C57BL/6J mice (male, 3 months of age) received three mild impacts (mTBI) or underwent sham procedures (sham). Mice were sacrificed 6 months later and brains were fixed for immunohistochemistry with an antibody against GFAP. Representative images were taken from cortex **(A)**, hippocampus **(C)**, and corpus callosum **(E)**, and GFAP immunoreactivity **(B,D,F)** was quantified as percentage of area occupied. Scale bar in **(A,C)** = 20 μm. Insert in **(A,C)** shows a high-magnification view of an astrocyte (arrow). *n* = 8 (sham) and 15 (mTBI) mice/group. Mean ± SEM. *P < 0.05; **P < 0.01, by *t* test.

In mice exposed to the repeat-impact mTBI, there was prominent astrogliosis throughout the brain. Astrocytes showed morphological characteristics of activation, including hypertrophic appearances with thick, densely labeled processes, and large cell bodies (Figure 7). Semi-quantitative estimation revealed that the GFAP immunoreactivity was significantly increased in the ipsilateral cortex (under the impact), hippocampus (CA3 region), and corpus callosum in the mTBI group compared with the sham group (Figure 7).

### Axonal degeneration after repetitive mTBI

**Figure 8:**
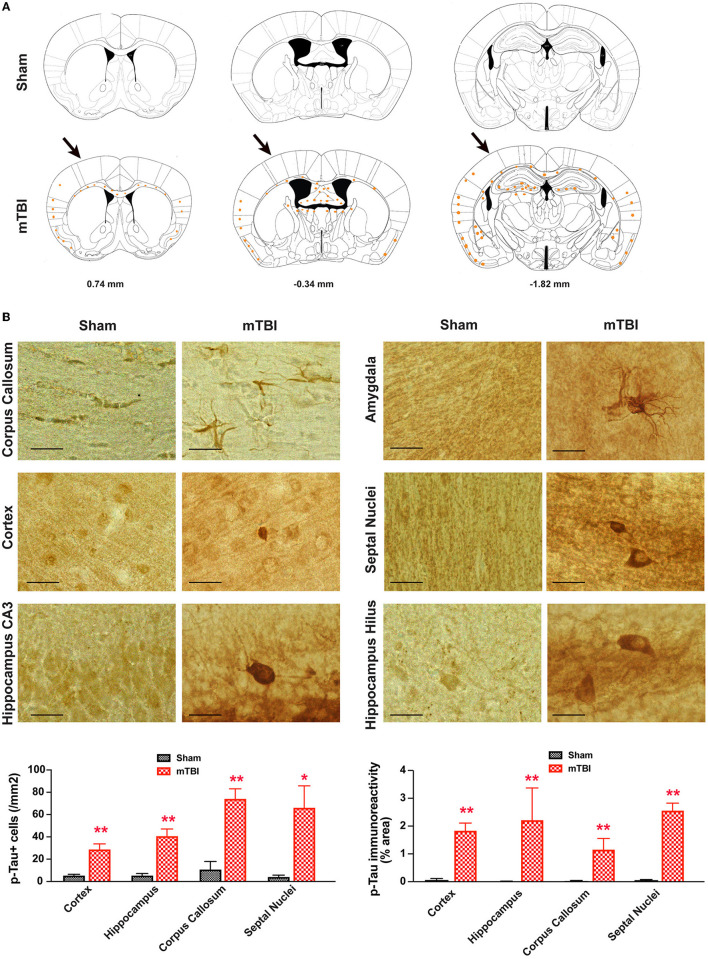
**Prominent p-Tau immunoreactivity after repetitive mTBI**. Wild-type C57BL/6J mice (male, 3 months of age) received three mild impacts (mTBI) or underwent sham procedures (sham). Mice were sacrificed 6 months later and brains were fixed for immunohistochemistry with an antibody against phospho-Tau (p-Tau, AT8). **(A)** Schematic diagrams of brain sections adapted from the mouse brain atlas (26) represent the approximate antero-posterior levels (to Bregma) where consistent neuropathological alterations were observed in mTBI (bottom) compared with sham (top). Note less p-Tau immunopositive cells in the contralateral side. The arrows in the bottom panel show approximate point of impact. **(B)** Representative images obtained from ipsilateral side of mTBI showing p-Tau immunoreactive cells in the corpus callosum, amygdala, hippocampus, and septal nuclei. Notice the weak or absence of p-Tau immunoreactivity in sham group. Scale bar = 20 μm.

In sham treated mice, weak p-Tau immunoreactivity was observed in a few, scattered cells in the brain; in contrast, stronger p-Tau immunoreactivity was observed consistently from 0.74 to −1.82 mm to Bregma (26) (Figure 8A), in regions including the ipsilateral corpus callosum, cortex, hippocampus, septal nucleus, and amygdala (Figure 8B). In addition, weaker p-Tau immunoreactivity was also observed in the contralateral side (Figure 8A).

### Reduced p-CREB immunoreactivity after repetitive mTBI

**Figure 9:**
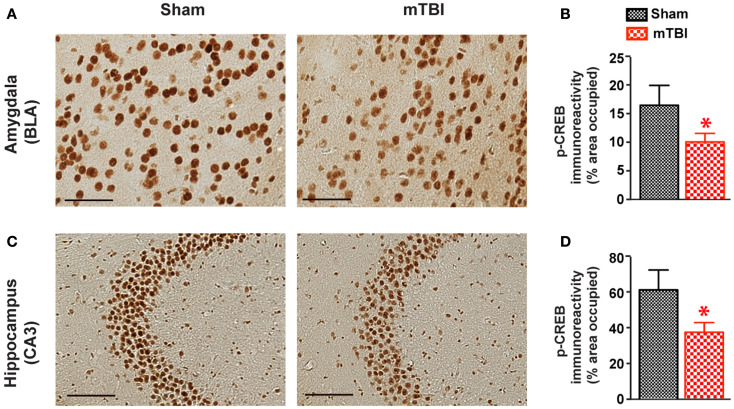
**cAMP responsive element binding protein phosphorylation was significant reduced after repetitive mTBI**. Wild-type C57BL/6J mice (male, 3 months of age) received three mild impacts (mTBI) or underwent sham procedures (sham). Mice were sacrificed 6 months later and brains were fixed for immunohistochemistry with an antibody against p-CREB. Representative images were taken from amygdala **(A)** and hippocampus **(C)**, and p-CREB immunoreactivity **(B,D)** was quantified as percentage of area occupied. Scale bar in **(A,C)** = 20 μm. *n* = 8 (sham) and 15 (mTBI) mice/group. Mean ± SEM. *P < 0.05, by *t* test.

To identify the potential molecular mechanisms responsible for the observed cognitive deficits, we examined the activation of CREB signaling. The CREB signaling pathway plays a critical role in neuronal survival and in memory formation and cognitive function (38, 39). Previous studies have shown this pathway is altered in moderate to severe TBI (40, 41), but its role in mTBI has not been investigated. We therefore performed immunohistochemistry and compared p-CREB immunoreactivity in both mTBI and sham brains. There was a significant reduction of p-CREB immunoreactivity in the hippocampus (CA3 neurons) and amygdala (the central nucleus, CeA) of the mice receiving mTBI compared with sham animals (Figure 9), suggesting the CREB signaling pathway is compromised after mTBI.

## Discussion

In this study, we have developed and characterized a mouse model of repetitive mTBI using an electromagnetic stereotaxic impact device. We show that animals subjected to three repetitive mTBI with an inter-impact interval of 24 h displayed long-term (6 months after injury) cognitive impairment, accompanied by prominent pathological changes. These results demonstrate that repetitive mTBI in mice leads to long-term consequences that resemble those reported in people with repeat exposure to mTBI and CTE.

A major challenge for developing experimental models of mTBI is to replicate the long-term cognitive dysfunction, an important feature of human mTBI patients (20). Although most published mTBI models have demonstrated the ability to produce immediate or short-term behavioral deficits, how long the cognitive dysfunction lasts is unknown, since few studies made long-term observations (beyond 1 month) (17, 18). In this study, we employed two frequently used cognitive tests and demonstrated that animals subjected to repetitive mTBI displayed cognitive deficits at 2 (RAWM) and 6 months after injury (contextual fear conditioning).

Cognitive deficits are a common problem seen in patients with repetitive mTBI (21). The hippocampus is an important brain region involved in learning and memory and is susceptible to mild head injury (21). In our model, the repetitively injured mice displayed behavioral deficits in hippocampus-dependent learning paradigms such as RAWM and contextual and cued fear conditioning (33), suggesting that hippocampal function is compromised in the mice in our model. The impacts in our model were applied to the temporal side of the mouse head and thus probably caused damage in the hippocampus and the cortex. The behavioral deficits were observed only in repetitively injured mice that received three impacts but not in the mice that received only a single impact, suggesting that long-term behavior deficits are attributed to the accumulative effects of multiple impacts.

Interestingly, mice exposed to repeated head impacts in our model showed a transient increase in locomotor activity (Figure 3). This is in agreement with observations from athletes who have experienced repeated mild concussive injuries, and often develop secondary problems with attention. These attention deficits are associated with hyperactivity (42). Hyperactivity has been observed after repetitive mTBI in mice in a weight drop model (43), suggesting that the increased locomotor activity is a common feature after mTBI and provides additional validation of our model.

Pathologically, we observed several changes following repetitive mTBI injury in our model: (1) the absence of gross morphological damage to the brain or obvious loss of neural tissue beneath the point of impacts. (2) Mice subjected to repetitive mTBI show prominent astrogliosis, as shown by increased GFAP immunostaining. (3) Perhaps of greatest interest is the increased immunoreactivity of p-Tau 6 months after the last injury. Tau is an intracellular, microtubule-associated protein that is highly enriched in axons. Hyperphosphorylation and pathological aggregation of Tau is indicative of axonal injury and a common feature of many neurodegenerative diseases with axonal degeneration. Therefore these observations provide strong evidence of traumatic axonal injury in our model, supporting the hypothesis that traumatic axonal injury is the primary pathology associated with adverse outcomes following mTBI (44). Furthermore, we consistently observed pathological changes in the hippocampus. This is not only in agreement with the results of behavioral tests, but also supports the notion that the hippocampus is especially vulnerable to mTBI. In addition, increased p-Tau immunoreactivity and astrogliosis could be pathological signs of CTE (45).

Mice receiving repetitive mTBI displayed reduced phosphorylation of CREB in the hippocampus. Since the CREB signaling pathway has been implicated in promoting the neuronal survival and in memory formation and cognitive function (38, 39), the reduction of CREB signaling may explain at least part of the robust cognitive deficits observed in behavioral tests in our model. Interestingly, the activation of the CREB pathway follows a bi-phasic pattern after TBI. In a fluid-percussion model producing a moderate TBI, CREB was activated at 30 min, peaked at 24 h, and returned to control level at 72 h after injury in the hippocampus (41). CREB signaling remained unchanged at 2 or 8 weeks post-injury, but was significantly decreased at 12 weeks after injury (40). How mTBI leads to reduced CREB signaling needs further investigation. Secondary injury mechanisms such as oxidative stress and excitotoxicity are both known to inhibit CREB phosphorylation and thus may play a role. It is also noteworthy that reduced immunoreactivity of p-CREB was observed in the absence of significant gross structural damage or neuronal cell loss, suggesting that compromised neuronal integrity may underlie long-term behavioral deficits.

GFAP is an extensively studied TBI biomarker and serum GFAP concentration is found to be predictive of death or poor outcome (46). Therefore, in this study we utilized a bioluminescence *in vivo* imaging technique in GFAP reporter mice. We have previously shown that the bioluminescence signal in the GFAP-luc mice correlates significantly with astrogliosis assessed by immunohistochemistry and bioluminescence imaging of these reporter mice is more sensitive in detecting astrogliosis than immunohistochemistry in a model of autoimmune encephalomyelitis (22, 23). We show here that the GFAP-luc reporter gene is also responsive to mTBI and can be used to monitor the initial astroglial response to mild brain injury and maybe more importantly, could be used to monitor the efficacy of experimental treatments for mTBI.

In our model the impact is delivered non-invasively and directly to the mouse head, without surgical intrusions. Our choice of the impact force (4 m/s) produces no skull fracture, nor external damage to the brain tissue. In addition, the impacts do not induce significant motor deficits or anxiety, indicating the injury did not affect the animals’ well being and thus allows a reliable assessment of the cognitive consequences of repetitive impacts to the brain (47). Our setup allows for accurate delivery of repetitive mild injuries to the same subject. The procedure is simple and rapid and can be performed without craniotomy or other manipulations to the head or skull. Thus our model is suitable for medium- to high-throughput screening of therapeutic compounds in a closed head, mild brain trauma.

In conclusion, we have developed a mouse model of repetitive mTBI with long-term behavioral and pathological sequelae, which resemble those observed in human mTBI patients. The model can be used to study the long-term neurological and pathological consequences of different numbers and frequencies of mild head injuries, and the molecular mechanisms of repetitive concussive injury to the brain. Our model may also be suitable for evaluating potential therapeutic interventions for mTBI.

## Conflict of Interest Statement

The authors declare that the research was conducted in the absence of any commercial or financial relationships that could be construed as a potential conflict of interest.

## Footnotes

^1^ http://www.afasci.com/

^2^ http://empress.har.mrc.ac.uk/viewempress/pdf/ESLIM_010_001.pdf
