# Corrigendum: Long-Term Cognitive Impairments and Pathological Alterations in a Mouse Model of Repetitive Mild Traumatic Brain Injury

**DOI:** 10.3389/fneur.2022.730576

**Published:** 2022-06-29

**Authors:** Jian Luo, Andy Nguyen, Saul Villeda, Hui Zhang, Zhaoqing Ding, Derek Lindsey, Gregor Bieri, Joseph M. Castellano, Gary S. Beaupre, Tony Wyss-Coray

**Affiliations:** ^1^Department of Neurology and Neurological Sciences, Stanford University School of Medicine, Stanford, CA, USA; ^2^Center for Tissue Regeneration, Repair and Restoration, VA Palo Alto Health Care System, Palo Alto, CA, USA

**Keywords:** mild traumatic brain injury, long-term, neurobehavior, bioluminescence, astrogliosis

In the original article, there was a mistake in Figure 8B as published. In the left bottom panel of Figure 8B, the image representing hippocampal CA3 of sham controls was from an animal with mTBI. The error was introduced while making figures. The corrected Figure 8 appears below showing images from a different experiment that confirmed the original findings, with additional quantitative analysis showing the difference between sham and mTBI animals.

**Figure 8:**
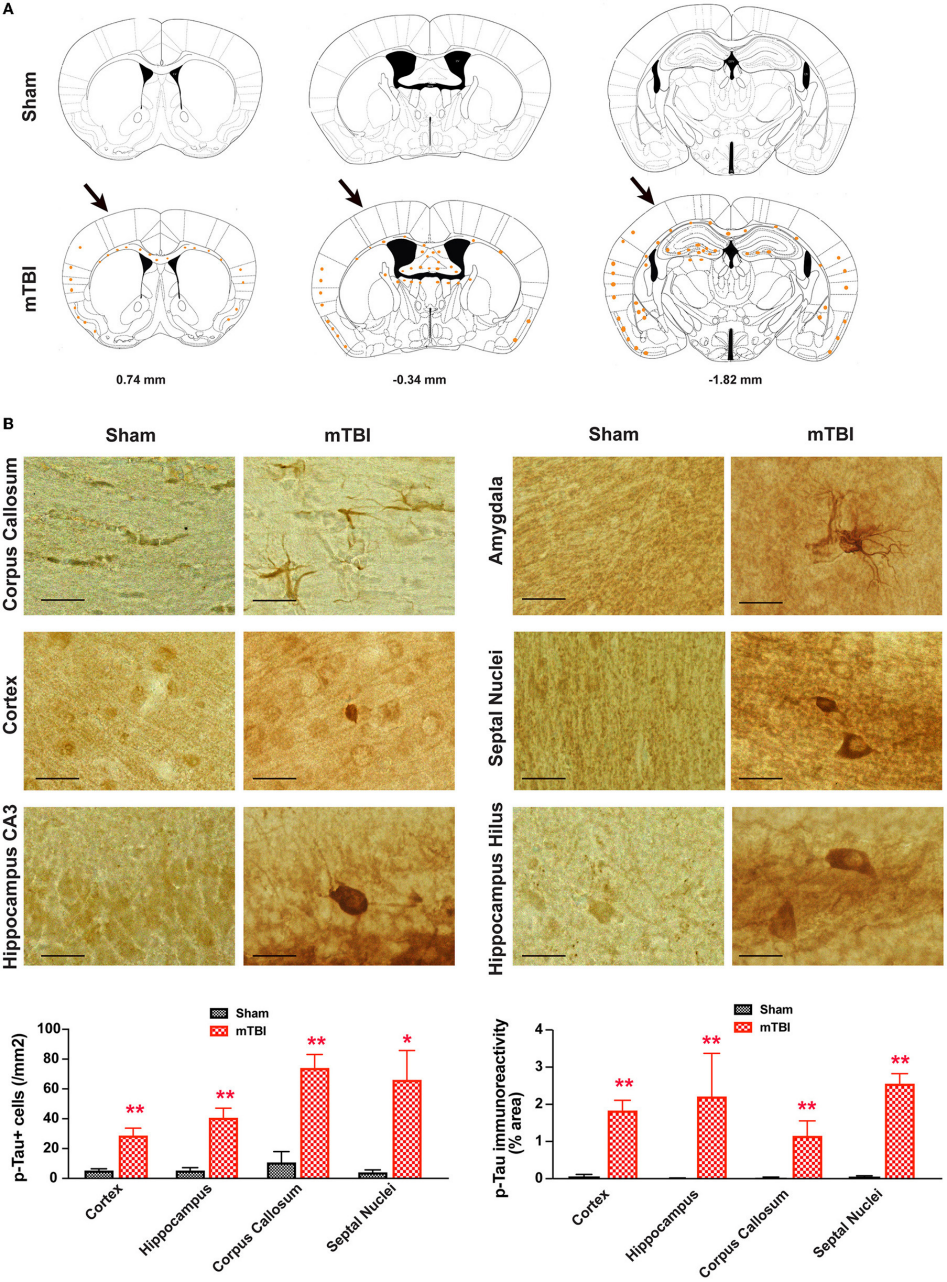
Prominent p-Tau immunoreactivity after repetitive mTBI. Wildtype C57BL/6J mice (male, 3 months of age) received three mild impacts (mTBI) or underwent sham procedures (sham). Mice were sacrificed 6 months later and brains were fixed for immunohistochemistry with an antibody against phospho-Tau (p-Tau, AT8). **(A)** Schematic diagrams of brain sections adapted from the mouse brain atlas (26) represent the approximate antero-posterior levels (to Bregma) where consistent neuropathological alterations were observed in mTBI (bottom) compared with sham (top). Note less p-Tau immunopositive cells in the contralateral side. The arrows in the bottom panel show approximate point of impact. **(B)** Representative images obtained from ipsilateral side of mTBI showing p-Tau immunoreactive cells in the corpus callosum, amygdala, hippocampus, and septal nuclei. Notice the weak or absence of p-Tau immunoreactivity in sham group. Scale bar = 20 μm. p-Tau immunoreactivity of ipsilateral side (bottom panels) was quantified as numbers of p-Tau+ cells (left panel) or percentage of area occupied (right panel) from different brain regions. Mean ± SEM. ^*^P <0.05; ^**^P <0.01, by *t*-test.

The authors apologize for this error and state that this does not change the scientific conclusions of the article in any way. The original article has been updated.

## Publisher's Note

All claims expressed in this article are solely those of the authors and do not necessarily represent those of their affiliated organizations, or those of the publisher, the editors and the reviewers. Any product that may be evaluated in this article, or claim that may be made by its manufacturer, is not guaranteed or endorsed by the publisher.

